# Alterations in expression of miRNA 497 and long non-coding RNAS (*XIST–TSIX*) and its significant role in colorectal cancer prediction

**DOI:** 10.1038/s41598-025-90110-3

**Published:** 2025-03-03

**Authors:** Mohamed Hassan Abd El-Magied, Amal Fawzy, Marwa Mohsen Mostafa, Ghada Nabil Elnaggar, Said Salama Moselhy, Mostafa Mohamed Elhady

**Affiliations:** 1 Clinical Pathology Department, National Cancer Institute, Cairo University, Cairo, Egypt; 2 Cardiovascular Hospital, Ain Shams University Hospitals, Ain Shams University, Cairo, Egypt; 3 Biochemistry Department, Faculty of Science, Ain Shams University, Cairo, Egypt

**Keywords:** Colorectal cancer, miRNA 497, Long non-coding RNAS, Diagnosis, Cancer screening, Biochemistry

## Abstract

Colorectal cancer (CRC) is a common type of malignancy in Western nations with high incidence related to different factors as genetic, foods and pollution. Long non-coding RNAs (LncRNAs) play a significant role in cellular processes, oncogensis and can be used as biomarkers for cancer progression. The rationale of this study was to quantify the expression levels of miRNA 497 and LncRNAs (XIST–TSIX) as a sensitive and accurate markers for CRC diagnosis and correlated with serum FOXK1, CA19.9 and CEA compared with normal subjects. This study was carried outon100 participants, they were divided into two equal groups: Group (1): Patients were diagnosed with CRC and Group (2): Normal subjects as control. Tumor size, type, TNM staging, differentiation, levels of FOXK1and, CEA, CA19.9 were evaluated in serum. The RNA was extracted from the tissue of CRC patients for quantification expression of miRNA 497 and LncRNAs (XIST and TSIX) using qRT-PCR. Data obtained showed that, the expression levels of tissue miRNA 497, XIST, TSIX in combination with serum FOXK1, CA19.9 and CEA are good confirmatory non-invasive markers for CRC diagnosis. Sensitivity and specificity tests showed higher AUC values of miRNA 497 + XIST + TSIX + FOXK1 significantly than those of CA19.9 + CAE. It was concluded that, a rigorous assessment of these parameters could facilitate the discovery of non-invasive biomarkers for the early detection and prognosis of CRC, ultimately enhancing the protocols for early treatment decision-making.

## Introduction

Cancer represents a prominent contributor in universal mortality, estimating almost one in every six deaths. It was reported that there were 20 millions new cancer cases and 9.7 millions cancer-related deaths worldwide. The cancer burden will rise by roughly 77% by the year 2050, affecting health systems, individuals, and communities^1^.

The impact of cancer extend beyond individual health, tendering considerable provocations for families and society as a whole due to decreased productivity resulting from the premature loss of individuals annually^2^.

The colorectal cancer (CRC) is rising in developing countries as they foster Western dietary patterns. Notably, there is a growing incidence among individuals under 45 years of age^3^. Although mortality rates for CRC have declined in recent years, this amendment is beginning to steadiness. Countries that have implemented effective barring and remediation strategies showed a decreased in mortality rates^4^.Over the last ten years, uplifting in research have enhanced our understanding of the disease, extended treatment options, and perfected overall survival rates. The life expectancy can be attributed to systemic therapies, treatment protocol, and surgical intervention^5^.

Biomarkers play a paramount role in the healthcare by exhibiting fundamental acquaintance concerning disease diagnosis, prognosis, treatment responses, and the progression of personalized medicine. Its significant measures expedite early detection and timely intervention, ultimately enhancing patient outcomes and reducing the costs. Furthermore, biomarkers assist in tailoring treatment interventions by predicting disease and enabling customized therapeutic strategies^6^.

In addition, biomarkers are important in follow up disease progression, adjusting treatment protocols, and recognizing early signs of recurrence, share significantly to drug development and clinical trials by pinpointing suitable patient populations and accelerating the approval process^7^.

In CRC, carcinoembryonic antigen (CEA) is the most prevalent serum biomarker for disease detection. CEA is a protein produced during fetal development and by some cancer cells. In healthy subjects, CEA levelwas minimal level; however, it was elevated in patients suffering from CRC^8^. It is serious to warrant that CEA is not exclusively index of CRC, as elevated levels can also exist in other circumstances, such as colonic inflammation. Nevertheless, CEA is a valuable biomarker for monitoring CRC patients for potential recurrence or disease progression^9^.

Another serum biomarker is carbohydrate antigen 19-9 (CA 19-9). This protein is produced by the pancreas and some cancerous tissues, particularly those in carcinoma. CA 19-9 is normally found in limited quantity in the general population but elevated levels can be observed in patients with CRC particularly in advanced stage. However, CA 19-9 is not specific to CRC and elevated in other conditions such as pancreatitis. It is used for monitoring the recurrence or progression of CRC^10^.

The rational of current study to identify a more sensitive, specific biomarker for CRC.

The human transcriptome was significantly related to its roles in regulatory non-coding RNAs, such as microRNAs (miRNAs) and long non-coding RNAs (LncRNAs), in both physiological and pathological conditions, including cancer. MiRNAs are small non-coding RNAs, play crucial roles in various cellular functions by regulating gene expression at the post-transcriptional level. Their stable expressions in cancer tissues and ease of detection have positioned them as potential candidates for cancer prognosis^11^.

Among these, microRNA497 has emerged as a key player in cancer biology, particularly in inhibiting angiogenesis and metastasis by targeting vascular endothelial growth factor. Its expression is notably reduced in numerous tumor types, suggesting its function as a tumor suppressor. However, elevated levels of miR-497 have been observed in certain cancers, indicating a complex role in tumorigenesis^12^. LncRNAs, also play significant regulatory roles in cellular processes and have been implicated in cancer development. They can be released into biological fluids; forming stable circulating LncRNAs that may keep pledge as valuable biomarkers for cancer revealing^13^. Notably, LncRNAs such as XIST and TSIX are involved in X chromosome inactivation, a critical process for proper cellular function and development. These LncRNAs also influence tumor pathogenesis by regulating apoptosis, proliferation, invasion, and migration of cancer cells, highlighting their potential as targets for further research in cancer treatment and understanding^14^.

The fork head box protein (FOXK1) was demonstrated to play a role in the occurrence, development, invasion, and spread of number of cancers, including gastric, colon^15^, glioma, and oesophageal cancer^16^. Despite the insights provided by these studies, they are constrained by limitations like small sample sizes and contentious findings. Consequently, to enhance our comprehension of the connection between non-coding RNAs and the diagnosis of colorectal cancer (CRC) patients, this research was initiated.

This study targeting to quantify the expression levels of miRNA 497 and long non-coding RNAS (XIST–TSIX) and correlated with serum biomarkers in CRC patients compared with normal subjects for precision and accuracy CRC diagnosis.

## Subjects and methods

### Study participants

The study followed the guidelines of the 1964 declaration of Helsinki, with approval from the Institutional Review Board at NCI (Approval# CP2301-503-011) and informed consent obtained from all participants. This study was carried out on 100 participants that were divided into two equal groups: **Gr1**, consisting of recently diagnosed CRC patients from the outpatient clinics of the National Cancer Institute (NCI) hospital, Cairo University and **Gr2**; comprising 50 apparently healthy individuals recruited from relatives of CRC sufferers. Demographic data as age, gender, smoking habits, tumor size, type, TNM staging, differentiation, and family history of cancer were collected from their records at the Biostatistics and Cancer Epidemiology Department, NCI.

### Samples collection

Blood samples (5 mL) were collected from each subject prior to surgical operations using vacutainer tubes after a 10 min rest under complete aseptic precautions. The samples were allowed to clot for 30 min in a plain collection tube, and then centrifuged at 4000 rpm for 10 min to obtain sera, which were stored at − 80 °C until analysis. Tissue samples were collected from each patient and examined to confirm diagnoses and categorize the specimen as benign or malignant. Tissue samples from excised safety margins were considered normal healthy tissues.

### Biochemical investigations

The serum levels of CEA and CA19.9 were measured before a surgical procedure using a chemiluminescent enzyme immunoassay on the Architect^®^ Analyzer according to guidelines provided by the manufacturer (Abbott Laboratories). Additionally, enzyme immunoassay (EIA) kit provided by Fujirebio Diagnostics was used for estimating the levels of FOXK1 (Catalog No. E7115Hu).

### Molecular analysis

The RNA was isolated from tissue tissue samples obtained (CRC and normal) by using miRNeasy tissue Mini Kit for purification of total RNA manufactured by QIAGEN Sample & Assay Technologies (Catalog No. 217004). The isolated RNA was then served as a template for synthesis of complementary DNA (cDNA) through using miRCURY LNA RT Kit (Qiagen, Hilden, Germany) (Catalog No. 339340) for miRNA 497 passay and RT2 LncRNA First Strand Kit (Qiagen, Hilden, Germany); (Catalog no. 330701) for long non-coding RNAS (XIST–TSIX). Following the manufacture guidelines, a qRT-PCR assay was carried out using miRCURY LNA SYBR^®^ Green PCR Kit and the miRCURY LNA miRNA PCR Assay (Qiagen, Hilden, Germany); (Catalog No. 339320). For miRNA 497 passay and EnTurbo^™^ SYBRGreen PCR SuperMix (High ROX Premixed); (Catalog No. EQ013) for long non-coding RNAS (XIST–TSIX). The relative expression levels of miRNA 497 and long non-coding RNAS (XIST–TSIX) were determined as 2^−ΔΔCT^ values, normalized to miR-103a-3p and β-actin respectively.

### Statistical analysis

Parametric numerical data is typically described using the mean and standard deviation, while non-parametric numerical data is described using the median and range. Non-numerical data is presented using frequency and percentage. The Student T test is used to compare the means of two study groups, while ANOVA is employed for comparing more than two groups’ means. The Mann Whitney test and Kruskal–Wallis test are utilized for non-parametric variables between two and more than two study groups, respectively. The Chi-Square test examines the relationship between two qualitative variables, while Fisher’s exact test is used when the expected count is less than 5 in over 20% of cells. Correlation analysis assesses the strength of association between two quantitative variables using correlation coefficients. The ROC Curve evaluates the sensitivity and specificity of diagnostic measures, with specific AUC value categories. Regression analysis, including logistic and ordinal regression, is used to predict risk factors using generalized linear models.

## Results

### Clinico-pathological findings of participants

The represented data in Table 1 revealed that adenocarcinoma was the most common type of cancer in CRC participants, accounting for 82.0% of cases, followed by mucinous adenocarcinoma at 16.0%, and adenosquamous carcinoma at 2.0%. In terms of staging, stage II was the most predominant at 42.0%, followed by stage I at 34.0%, with stages III and IV being less common at 18.0% and 6.0% respectively. Regarding the grades of CRC cases, Grade II was the most common at 70.0%, followed by Grade I at 16.0% and Grade III at 14.0%. The colon was the most common site of tumors at 42.0%, while tumors in the rectum accounted for 16.0% of cases. Lymphadenopathy was present in 22.0% of cases and absent in 78.0%. Metastasis was present in 6.0% of cases, with the majority showing no signs of metastasis at 94.0%. Among cases with metastasis, 66.7% had metastasis to the liver and 33.3% had metastasis to the fallopian tube (Table 1).

**Table 1:** Individual characteristics in all studied groups.

Clinico-pathological features for CRC patients		
Type of cancer	N	%
Adenocarcinoma	41	82.0%
Mucinous Adenocarcinoma	8	16.0%
Adenosquamous carcinoma	1	2.0%
Stage		
Stage I	17	34.0%
Stage II	21	42.0%
Stage III	9	18.0%
Stage IV	3	6.0%
Early stage (I&II)	38	76%
Late stage (III&IV)	12	24%
Grades		
Grade I	8	16.0%
Grade II	35	70.0%
Grade III	7	14.0%
Sites		
Colon	42	84.0%
Rectum	8	16.0%
Lymphadenopathy		
Absent	39	78.0%
Present	11	22.0%
Metastasis		
Absent	47	94.0%
Present	3	6.0%
Site of metastasis		
Liver	2	66.7%
Fallopian tube	1	33.3%

Qualitative data are represented as the number of cases and percentage (%).

### Biochemical and molecular findings

**Fig. 1:**
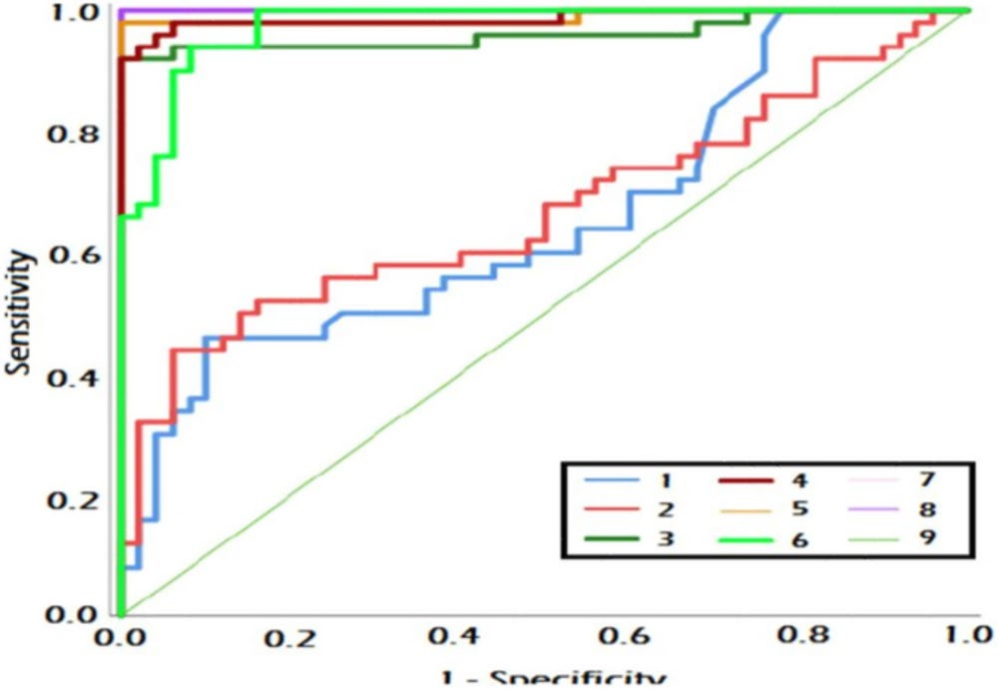
ROC of combined studied markers for discrimination between CRC and control group.1, CA19.9; 2, CA19.9 + CEA; 3, CA19.9 + miRNA 497; 4, CA19.9 + XIST expression level; 5, CA19.9 + TSIX expression level; 6, CA19.9 + FoxK1; 7, miRNA 497 expression level + XIST expression level + TSIX expression level + FoxK1; 8, CA19.9 + miRNA 497 expression level expression level + XIST expression level + TSIX expression level + FoxK1and 9, reference line.

**Fig. 2:**
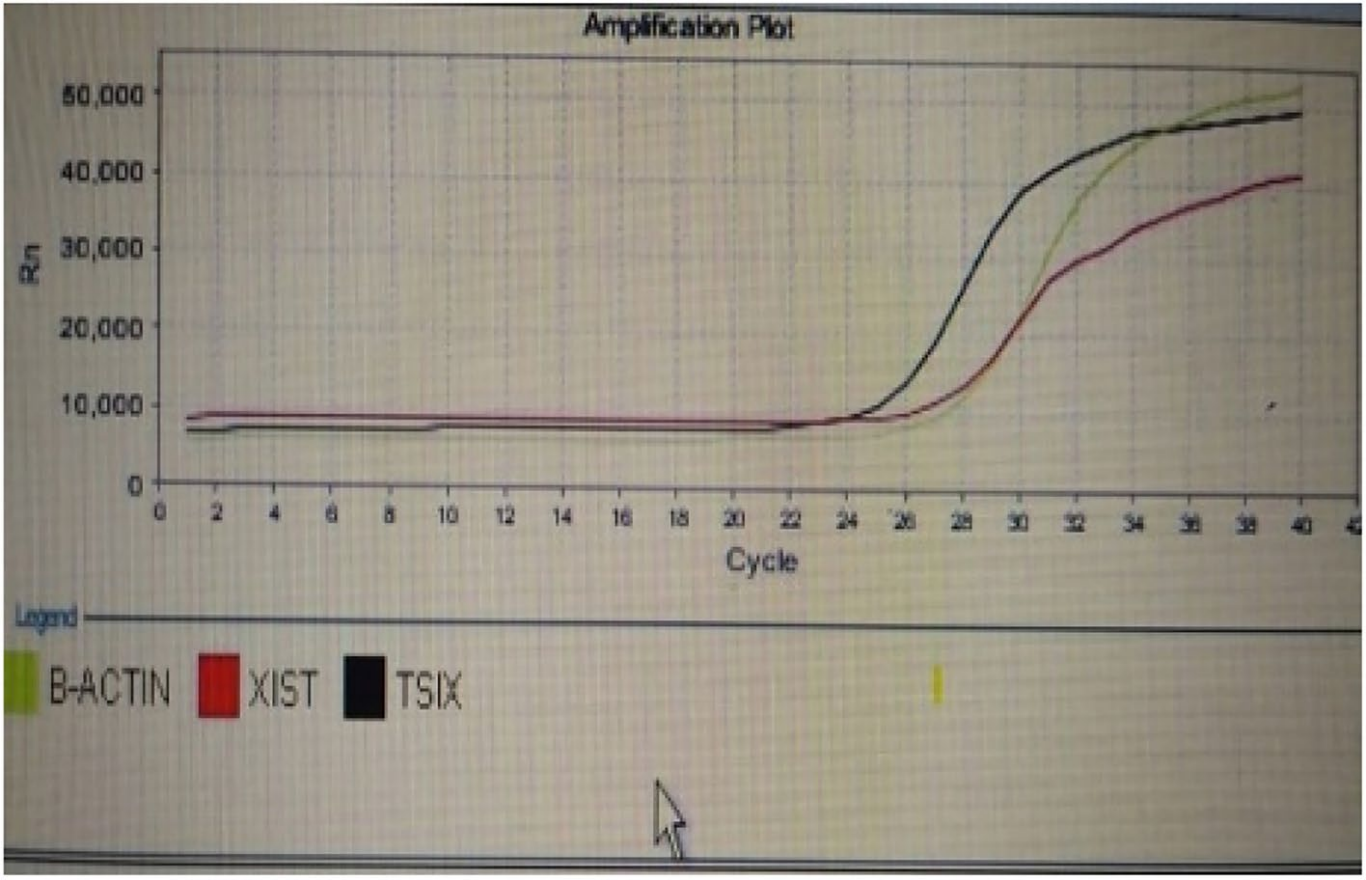
The XIST long non coding RNA vs TISX in CRC.

**Fig. 3:**
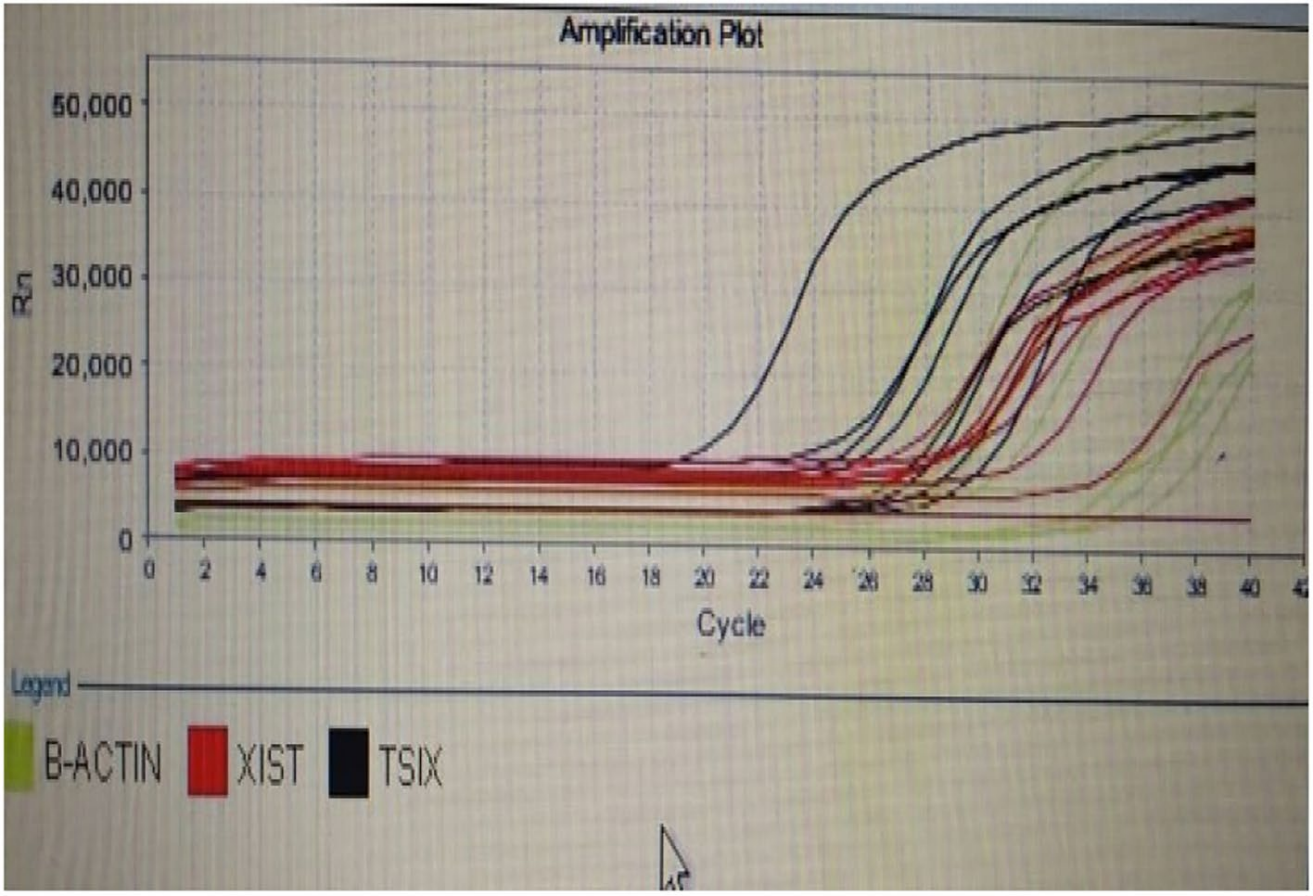
The XIST long non coding RNA vs TISX in CRC.

**Fig. 4:**
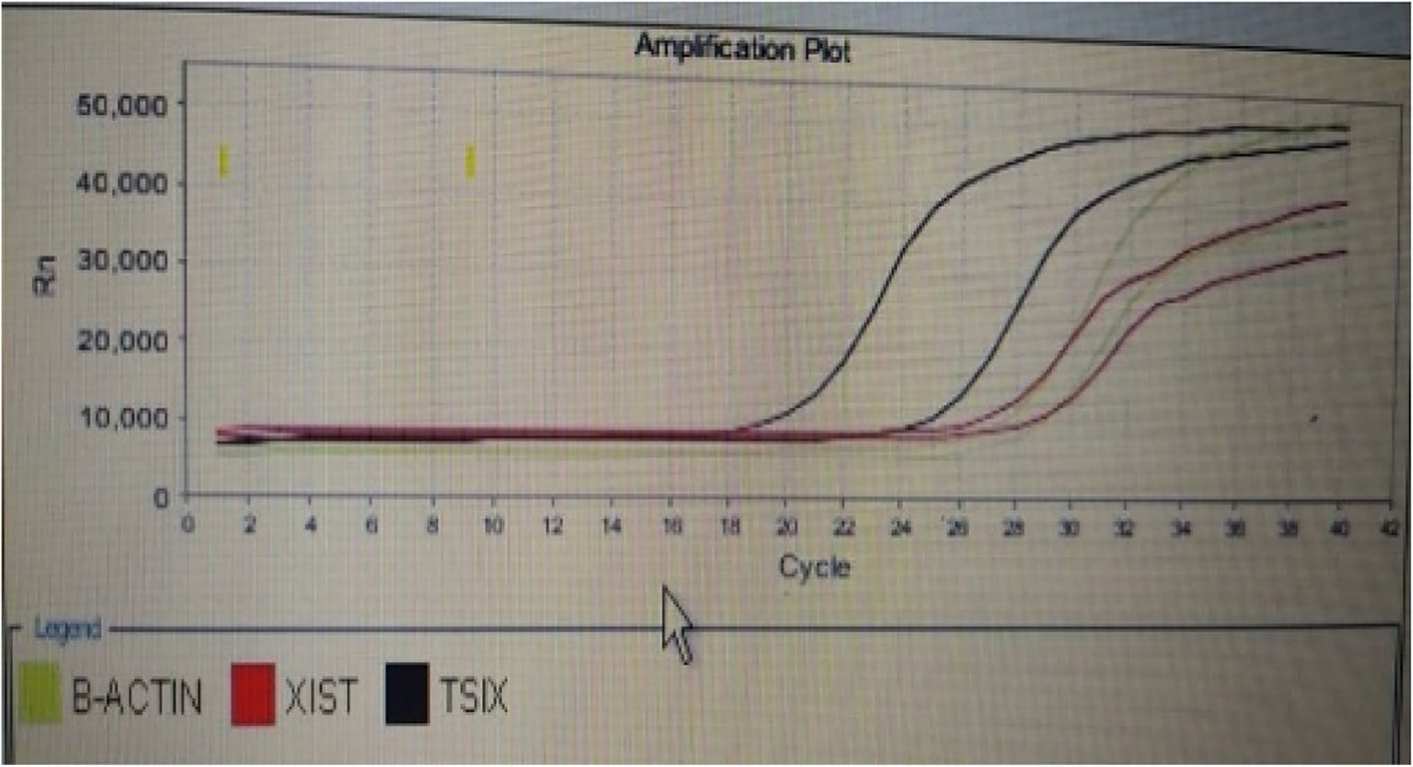
Micro RNA 497 in CRC.

**Fig. 5:**
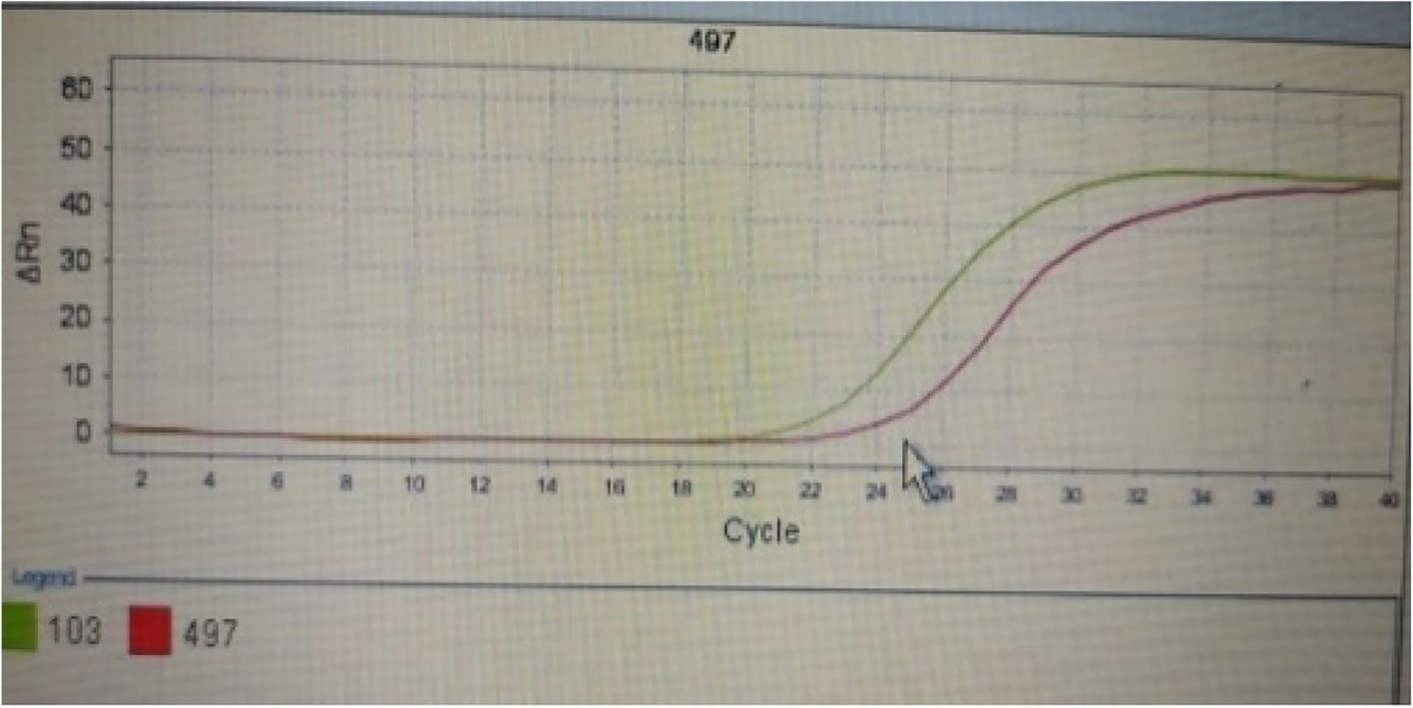
Micro RNA 497 in CRC.

The combination of measured parameters demonstrated excellent discriminatory ability, surpassing the performance of CA19-9 and CA19-9 combined with CEA. All combinations exhibited significantly higher AUC values compared to CA19-9 and CA19-9 combined with CEA. Additionally, the AUC values of miRNA 497 + XIST + TSIX + FOXK1and CA19.9 + miRNA 497 + XIST + TSIX + FOXK1were significantly higher than those of CA19.9 + FOXK1as shown in Fig. 1. The represented data in Table 2 showed that there was a non-significant increase in the level of CEA in CRC patients compared to the control group. However, there was a significant increase in the levels of CA19.9 and FoxK1 in CRC patients compared to the normal group. The median expression level of miRNA 497 was significantly downregulated in CRC patients compared to the control group. The long non-coding RNA XIST gene was significantly over-expressed in CRC patients (Figs. 2, 3, 4, and 5), while the TSIX gene was significantly downregulated in CRC patients compared to the control group (Table 3). There were no notable disparities observed in serum and tissue levels of examined parameters based on gender, stage, grade, tumor site, lymphadenopathy, and metastasis in CRC patients.

**Table 2:** Comparison of serum levels of CEA, CA19.9 &FOXK1 among studied groups.

	Control	CRC	*p*
Biochemical parameters			
Serum CEA (ng/ml)			
Median (min–max)	4.4(1.1–51.1)	8.4(1.7–200.4)	0.008*
Serum CA19.9 (U/ml)			
Median (min–max)	1.55(0.4–9.3)	2.05(0.4–86.5)	0.170
Serum FOXK1 (ng/ml)			
Median (min–max)	3.03(1.5–5.9)	6.1(3.4–23)	< 0.001*

CEA, carcinoembryonic antigen; CA 19.9, carbohydrate antigen 19-9; FOXK1, fork head box protein; CRC, colorectal cancer.

*indicates a statistically significant difference.

**Table 3:** Comparison of tissue gene expression of miRNA 497, XIST & TSIX among studied groups.

	Control	CRC	*p*
Molecular parameters			
Tissue miRNA 497 expression			
Median (min–max)	1.39(1–17.4)	0.387(0.002–2.271)	< 0.001*
Tissue XIST expression			
Median (min–max)	0.607(0.045–1)	4.53(0.5–187)	< 0.001*
Tissue TSIX expression			
Median (min–max)	1.455(1–12.9)	0.29(0.004–1.7)	< 0.001*

XIST, X inactivation specific transcript; TSIX, antisense to xist; CRC, colorectal cancer.

*indicates a statistically significant difference.

It was found that, FOXK1displayed the best area under the curve (AUC) at 0.974, followed by CA19.9 at 0.655 and CEA at 0.580. In terms of molecular targets, TSIX showed a superior AUC at 0.989 compared to XIST at 0.984 and miRNA 497 at 0.963as shown in Table 4. The combination of measured parameters demonstrated excellent discriminatory ability, surpassing the performance of CA19-9 and CA19-9 combined with CEA.

**Table 4:** The diagnostic validity of the estimated parameters in discrimination between CRC cases and control group.

	Tissue			Serum		
	CEA	CA19-9	FOXK1	miRNA 497	XIST	TSIX
AUC	0.58	0.655	0.974	0.963	0.984	0.989
95% CI	0.467–0.692	0.547–0.763	0.947–1	0.92–1	0.961–1	0.968–1
*p*	0.17	0.008*	< 0.001*	< 0.001*	< 0.001*	< 0.001*
Cut off	> 2.5	> 8.0	> 3.75	≤ 1.0	> 1	≤ 1.0
Sensitivity (%)	46	50	98	94	96	98
Specificity (%)	64	64	90	74	86	84
PPV (%)	56.1	58.1	90.7	78.3	87.3	85.9
NPV (%)	54.2	56.1	97.8	92.5	93.4	97.7
Accuracy (%)	55	57	94	84	91	91

CEA, carcinoembryonic antigen; CA 19.9, carbohydrate antigen 19-9; FOXK1, fork head box protein; XIST, X inactivation specific transcript; TSIX, antisense to xist;AUC, area under ROC curve; CI, confidence interval; PPV, positive predictive value; NPV, negative predictive value.

*indicates a statistically significant.

### Correlation between the evaluated parameters

The analysis of various parameters revealed that CA19.9 was positive correlation with XIST and FOXK1, and a negative association with TSIX and miRNA 497. MiRNA 497 exhibited a positive correlation with TSIX and negative correlations with XIST and FOXK1. XIST was negative correlations with TSIX and positive correlation with FOXK1. Foxk1 showed negative correlations with miRNA 497 and TSIX, and positive correlations with CA19-9 and XIST. No other significant correlations were observed among the subjects studied, Table 5

**Table 5:** Correlations between targeted parameters among all studied subjects.

		miRNA 497	XIST	TSIX	FOXK1
Age	rs	− 0.043	− 0.004	− 0.066	0.060
	*p*	0.674	0.965	0.517	0.552
CEA	rs	− 0.071	0.077	− 0.102	0.100
	*p*	0.485	0.445	0.311	0.322
CA19-9	rs	− 0.283	0.216	− 0.303	0.196
	*p*	0.004	0.031	0.002	0.050
MiRNA 497	rs		− 0.705	0.646	− 0.637
	*p*		< 0.001	< 0.001	< 0.001
XIST	rs			− 0.693	0.711
	*p*			< 0.001	< 0.001
TSIX	rs				− 0.654
	*p*				< 0.001

CEA, carcinoembryonic antigen; CA 19.9, carbohydrate antigen 19-9; FOXK1, fork head box protein; XIST, X inactivation specific transcript; TSIX, antisense to xist.

The correlation was analyzed using Spearman’s rank correlation coefficient.

Univariable analysis showed that higher levels of CA19-9, XIST, and FOXK1, as well as lower levels of TSIX and miRNA 497, were linked to an increased risk of CRC susceptibility. However, in the multivariable analysis, only higher levels of XIST and FOXK1, along with lower levels of TSIX and miRNA 497, were identified as independent predictors of CRC susceptibility. It was suggested that, these specific biomarkers play a significant role in predicting CRC susceptibility as represented in Table 6.

**Table 6:** Regression analysis for prediction of CRC susceptibility.

	Univariable				Multivariable			
	*p*	OR	95% CI		*p*	OR	95% CI	
CEA	0.065	1.083	0.995	1.178				
CA19-9	0.016	1.032	1.006	1.058	0.619	0.993	0.619	0.993
XIST	< 0.001	2.149	1.174	6.814	< 0.001	2.930	1.021	3.930
TSIX	< 0.001	0.021	0.005	0.090	< 0.001	0.050	0.012	0.150
miRNA 497	< 0.001	0.091	0.042	0.199	0.020	0.092	0.020	0.102
FOXK1	< 0.001	3.415	2.212	5.272	0.019	1.993	1.919	2.993

CEA, carcinoembryonic antigen; CA 19.9, carbohydrate antigen 19-9; FOXK1, fork head box protein; XIST, X inactivation specific transcript; TSIX, antisense to xist; OR, odds ratio; CI, confidence interval.

## Discussion

Colorectal cancer (CRC) represents a multifactorial global health obstacle, being the third most frequently diagnosed cancer and the second main cause of cancer-related mortality. The precise etiology of CRC remains elusive, and early detection is often impeded by the lack of characteristic clinical signs. Notable disparities in incidence and mortality rates have been documented across various countries and regions, with an alarming increase in cases among younger populations in developed countries. Improving lifestyle, foods habits, and environmental influences are pivotal in the primary safeguard against colorectal cancer^17^.

In Egypt, CRCis approximately 6.5% of all malignant neoplasms. The prevalence is noteworthy, as 14.0% of colonoscopies performed reveal this condition. Especially concerning is the increased incidence of CRC between individuals aged 40 and younger^18^. Nevertheless, there is a scarcity of studies defining attitudes towards colorectal cancer examination and its implementation. The obligation to set guidelines for colorectal cancer screening remains mysterious, with evidence proposing that a substantial proportion of cases are diagnosed at advanced stages.

The current study analysis of CRC cohort referred that adenocarcinoma was the most common type, representing 82.0% of cases, followed by mucinous adenocarcinoma at 16.0%, and adenosquamous carcinoma at 2.0%. These results in accordance with previous study that reported a high prevalence of conventional adenocarcinoma among CRC patients^19^.

Regarding to staging, stage II emerged as the most prevalent, accounting for 42.0% of cases, while stage I represented 34.0%. Stages III and IV were noticed less frequently, comprising 18.0% and 6.0% of cases, respectively. This distribution of stages contradicts other similar studies which revealed that a relatively higher number of patients were diagnosed at the advanced stages, especially stage III^20^.

In terms of case grading, Grade II was the most commonly reported, representing 70.0% of cases, while Grade I and Grade III accounted for 16.0% and 14.0%, respectively. This grading pattern is harmonious with other research indicating a higher prevalence of lower histological grades among colorectal cancer (CRC) sufferers^21^^,^^22^.

The colon was designated as the primary lesion in 42.0% of the cases with rectal tumors accounting for 16.0%. This distribution also supports the literature which alludes to increased cases of colon cancer among CRC patients. Metastasis was ranked in 6.0% of cases and out of the cases with metastasis, liver metastasis was the most prevalent. This is in concordance with other studies that has corroborated a high prevalence of hepatic metastases in CRC patients^23^.

These variations when considering clinical and pathological may reflect the study draw from a single institution and the diversified ethnicity; creating a gap for more studies in order to fully appreciate these differences. The outcome of the present study also indicated a significant decrease in the expression of miRNA 497-5P in CRC patients as compared to control groups which is consistent with the previous studies reported by Kattan et al.^24^ and Hong et al.^25^.

Other studies have come out with the argument that as miRNA 497 was important in cell division, enhancement of apoptosis, and inhibition of cell migration and cell invasion of cancer cells took place thus pointing its likely tumor suppressive function. On the other hand, reduction of miRNA-497 would lead to increased growth, increased propensity to colonization, migration, invasion, and resistance to chemotherapy.

Data of FOXK1protein level revealed a significant elevation in CRC patients when compared to control subjects, corroborating the results reported by Wu et al. as well as earlier studies conducted by Wu et al.^15^ and Wang et al.^26^.

High level of FOXK1expression was observed in both CRC patients and cell lines, promoting cancer development through enhanced cell proliferation, as well as resistance to apoptosis, invasion, and metastasis. Especially, oncogenic activity of FOXK1was remarkably decreased as a consequence of inhibited motility and invasiveness of cancer cells suggesting its important participation in CRC progression characterized by high expression of FOXK1.

In terms of the median expression level of the XIST gene, higher levels were observed in the CRC participants than in the healthy volunteers. Most striking was the down-regulated expression of the TSIX gene in CRC tissue samples.

The XIST was shown to be overexpressed in CRC tissues, as reported by Wang et al.^26^ and Yang et al.^27^. Other studies done by Chen et al.^16^ and Song et al.^28^ also pointed to the increased level of expression of XIST in colorectal cancer tissues compared to that in normal tissues. Also, Song et al.^28^ suggesting that XIST may stimulate cell proliferation through inhibiting certain miRNA pathways. In addition, XIST has been associated with the presence of chemo-resistance and it was demonstrated in Xiao et al.^29^ that XIST was associated with 5-FU chemoresistance.

This means that targeting the XIST through silencing may provide the new therapeutic strategies to overcome 5FU resistance in the CRC patient. Furthermore, TSIX may also play a role in the regulation of XIST by ‘activating’ the RNA interference pathway in the sense that TSIX and XIST will base pair and form a long double stranded RNA which is then cleaved into small RNAs. The feasible mechanism of TSIX may also require modulation of the organization of chromatin and DNA methylation mark on XIST promoter.

The study explained how certain biochemical parameters can be used to differentiate between CRC patients and controls where FOXK1explains the highest AUC value of 0.974. For instance, TSIX had comparatively higher AUC value than that of XIST and miRNA 497 molecular targets. As for the two miRNAs, it has been identified that miRNA 497, XIST, TSIX, FOXK1, and CA19. Nine of them offered improved discriminative capability for distinguishing CRC patients from the control group, and higher accuracy compared to the efficacy of CA19-9 tested singularly or in combination with CEA.

The area under curve (AUC) values for the combined markers were significantly better than those for CA19.9 and FoxK1 when considered individually. These results suggest a powerful linkage between miRNA 497, XIST, TSIX, FoxK1, and CA19.9 with CRC, indicating potential biomarkers for the disease. It can improve the accuracy of CRC diagnosis and prognosis, increasing sensitivity and specificity.

The investigation revealed that CA19.9 is affirmative correlated with XIST and FoxK1, while it shows negative linkage with TSIX and miRNA 497. MiRNA 497 demonstrated a positive relationship with TSIX and negative associations with both XIST and FOXK1.

The miRNA 497 has a negative impact on FoxK1 expression and that lower levels of XIST expression are associated with higher levels of miR-497-5p^30^ and^26^. Additionally, significant negative correlations were observed between XIST and TSIX, as well as between FoxK1 and both miRNA 497 and TSIX. XIST and TSIX play crucial roles in the regulation of X chromosome inactivation (XCI), where the upregulation of XIST leads to the coating of the X chromosome, while TSIX acts as a negative regulator of XIST^31^.

The univariable analysis showed that increased levels of CA19-9, XIST, and FOXK1, as well as decreased levels of TSIX and miRNA-497, were linked to a greater risk of colorectal cancer (CRC). However, the analysis of multiple variables showed that only higher levels of XIST and FOXK1, along with lower levels of TSIX and miRNA 497, were independent predictors of CRC risk. Sefrioui et al.^32^ discovered that CA19-9 is a predictive factor for CRC patients with high baseline CA19-9 levels, while Chen et al.^16^ showed that lncRNA XIST expression was a prognostic factor for CRC patients.

These findings highlight the important estimated parameters play in the development of colorectal cancer, presenting new avenues for diagnosing and treating the disease. However, our study has some limitations that should be avoided in further research. Upcoming studies should be multicentric with a larger sample size for better verification of results. All pathological subtypes of CRC should be included as well as benign colorectal conditions. The studied markers should be compared with the patient’s follow up to assess disease free and overall survival.

Treatment approaches for CRC differ depending on the cancer’s stage and type. CRC surgery is linked to a wide range of complications that impact the procedure’s effectiveness as well as the general health and survival of the patients. The most common postoperative complications of colorectal surgery include surgical site infection (SSI), bleeding, anastomotic leakage, intraabdominal abscess and ileus. These issues require a precise diagnosis and have varying effects on the results^33^.

Post-colorectal surgery SSI is a significant healthcare concern since it is linked to a poor prognosis, higher fatality rates, longer hospital stays, and up to three times higher hospital expenses. The alpha-glycoprotein butyrylcholinesterase (BChE) is an enzyme found in most tissues, especially the liver. Studies show that low and declining BChE levels on the first and third day following colorectal surgery are associated with an increased risk for SSI, even after controlling known risk factors for the development of SSI^34^.

The majority of these postoperative complications often arise between the first week to the first month following surgery. As a result, these individuals require ongoing care throughout this time. The need for technology-based interventions to track surgical patients’ post-discharge health is growing due to the shortage of specialised staff and their heavy workload on the one hand, and the high expense of cancer patient care on the other^35^.

Modern technologies like Internet of Things (IoT), wireless sensor networks (WSN), and Internet of Medical Things (IoMT) have a major impact on the development of smart health monitoring systems and applications for the early detection of non-contagious diseases like cancer. These sensors are attached to the patient and continuously transfer vital health data to the medical team from anywhere and anytime^36^.

To prevent CRC-related mortality, early diagnosis and appropriate treatment are essential. Deep learning (DL), a subtype of artificial intelligence (AI), is one of the newest technologies for accomplishing this. DL protocols seem to optimise CRC diagnosis, by boosting diagnostic accuracy, enhancing the value of clinicians’ experience, reducing diagnostic variability among physicians, and optimising and/or expanding the extracted clinical data without conducting additional conventional diagnostic tests on the available diagnostic material (histologic samples, endoscopy images, etc.)^37^^,^^38^.

This approach will aid in validating results and elucidating the connections between these parameters and the risk of developing CRC. Furthermore, a rigorous assessment of these parameters facilitated the discovery of noninvasive biomarkers for the early detection and prognosis of CRC, ultimately enhancing the protocols for treatment decision-making.

## Data Availability

The datasets used and/or analyzed during the current study available from the corresponding author on reasonable request.
